# Fine Regulation of Neutrophil Oxidative Status and Apoptosis by Ceruloplasmin and Its Derivatives

**DOI:** 10.3390/cells7010008

**Published:** 2018-01-12

**Authors:** Ekaterina A. Golenkina, Galina M. Viryasova, Svetlana I. Galkina, Tatjana V. Gaponova, Galina F. Sud’ina, Alexey V. Sokolov

**Affiliations:** 1 Belozersky Institute of Physico-Chemical Biology, Lomonosov Moscow State University, Moscow 119234, Russia; golyesha@mail.ru (E.A.G.); gali-inimitable@yandex.ru (G.M.V.); galkina@genebee.msu.su (S.I.G.); 2 FGBU Hematology Research Centre, Russia Federation Ministry of Public Health, Moscow 125167, Russia; gaponova.tatj@yandex.ru; 3 FSBSI Institute of Experimental Medicine, St. Petersburg 197376, Russia; biochemsokolov@gmail.com; 4 Department of Fundamental Problems of Medicine and Medical Technology, Saint-Petersburg State University, St. Petersburg 199034, Russia; 5 Centre of Preclinical Translational Research, Almazov National Medical Research Centre, Saint-Petersburg 197371, Russia

**Keywords:** apoptosis, neutrophil, ceruloplasmin, superoxide, reactive oxygen species

## Abstract

Timely neutrophil apoptosis is an essential part of the resolution phase of acute inflammation. Ceruloplasmin, an acute-phase protein, which is the predominant copper-carrying protein in the blood, has been suggested to have a marked effect on neutrophil life span. The present work is a comparative study on the effects of intact holo-ceruloplasmin, its copper-free (apo-) and partially proteolyzed forms, and synthetic free peptides RPYLKVFNPR (883–892) and RRPYLKVFNPRR (882–893) on polymorphonuclear leukocyte (PMNL, neutrophil) oxidant status and apoptosis. The most pronounced effect on both investigated parameters was found with copper-containing samples, namely, intact and proteolyzed proteins. Both effectively reduced spontaneous and tumor necrosis factor-α (TNF-α)-induced extracellular and intracellular accumulation of superoxide radicals, but induced a sharp increase in the oxidation of intracellular 2′,7′-dichlorofluorescein upon short exposure. Therefore, intact and proteolyzed ceruloplasmin have both anti- and pro-oxidant effects on PMNLs wherein the latter effect is diminished by TNF-α and lactoferrin. Additionally, all compounds investigated were determined to be inhibitors of delayed spontaneous apoptosis. Intact enzyme retained its pro-survival activity, whereas proteolytic degradation converts ceruloplasmin from a mild inhibitor to a potent activator of TNF-α-induced neutrophil apoptosis.

## 1. Introduction

Human ceruloplasmin (CP, ferro:O_2_-oxidoreductase) is a glycosylated multi-copper ferroxidase that is synthesized primarily in the liver and is abundant in the plasma and interstitial fluid. It has been estimated to bind to 40–70% of total plasma copper in normal adults, and is the principle mode of copper storage in the body [1,2]. Additionally, the copper harbored by ceruloplasmin is available for uptake by tissues throughout the organism and, thus, one basic function of this “moonlighting protein” is Cu transport and delivery to tissues [3]. Copper ions are important for a large variety of the enzymatic activities of CP. Possessing ferroxidase activity, this enzyme plays an incontrovertible role in controlling iron turnover and homeostasis. Indeed, the oxidation of ferrous iron to its ferric state by CP is essential for the mobilization and release of iron from cellular stores, and for uptake by the circulating iron transport protein transferrin [4]. According to available data, CP prevents the participation of Fe^2+^ in reactions involving O_2_ and its reduction products, most importantly, H_2_O_2_, resulting in superoxide and hydroxyl radical formation, respectively [5]. 

Further to this direct antioxidant action, CP can affect protective enzymatic mechanisms, thus moderating immune responses [6]. Indeed, as the plasma concentration of this copper-containing protein increases from approximately 200–900 µg/mL in response to inflammation, CP refers to the positive acute-phase proteins. Its synthesis and secretion are also elevated in diabetes mellitus, cancer, cardiovascular and Alzheimer’s disease, rheumatoid arthritis, and during pregnancy [6]. 

CP has a direct effect on the function of human neutrophils. Particularly, it was reported that CP inhibits 5-lipoxygenase [7] and a number of serine proteases of leukocyte origin related to inflammatory and septic processes [8]. Anionic CP (pI 4.7) interacts with high affinity with cationic proteins of neutrophils, such as lactoferrin (LF) (pI 8–9) and myeloperoxidase (MPO) (pI 9–10) [9,10]. MPO is an enzyme found predominantly in neutrophils [11]. When it is secreted from activated neutrophils, the likelihood of its interaction with CP is high due to the increased concentration of the latter in inflammation loci [12]. During inflammation, CP is protective against oxidant production by MPO. The formation of a specific complex between CP and MPO has been described [13]. The entrance of the heme pocket (active center) of MPO interacts with the protease-sensitive loop between the 5th and 6th domains of CP (883–892) in the conventional 3D-model of the 2CP-MPO complex. The copper-free peptide RPYLKVFNPR, corresponding to sequence 883–892 of CP, exhibits potent inhibition of MPO; in contrast, CP subjected to limited proteolysis loses its efficiency as an inhibitor of MPO [13].

Despite a large amount of accumulating information, many gaps remain in the understanding of the nature of the effect of CP on neutrophil function. Research in this area is complicated by the fact that in inflammation loci, particularly in the synovial fluid of rheumatoid arthritis patients, a mixture of intact proteins and a greater or lesser degree of proteolyzed molecules must be considered [14]. 

Shifts in polymorphonuclear leukocyte (PMNL) redox status, as well as deviations in apoptosis propensity, have been considered to underlie several chronic inflammatory diseases. This indicates that studies on all possible elements involved in these regulatory mechanisms are important. This work is a comparative study of the in vitro effects of ceruloplasmin and its derivatives on neutrophil oxidative status and lifespan. The authors hypothesized that the fine-tuning of neutrophil activity by CP and its derivatives would in turn be adjusted under priming conditions similar to those seen during acute or chronic inflammatory events. The authors selected the cytokine TNF-α to model this in vitro.

## 2. Materials and Methods

### 2.1. Enzyme Isolation

CP was isolated from heparin-stabilized plasma of healthy donors and purified using ion-exchange chromatography with UNO-Sphere Q and affinity chromatography with neomycin-agarose [15]. Proteolyzed CP (CPprot) was obtained after limited proteolysis for 2 h at 37 °C with human thrombin (100:1, *w*/*w*). Thrombin was removed immediately after incubation using benzamidine-agarose chromatography. Molecular masses of CP and CPprot fragments were evaluated with the help of SDS-electrophoresis in high-molarity Tris-buffer system polyacrylamide gel [16]. SDS-PAGE of CPprot resulted in only fragments of 116, 72, 67, 52, and 19 kDa [14] (Table 1). Apo-CP was obtained by adding ascorbic acid (0.1 M), sodium azide (0.1 M), and EDTA (0.5 M) to 20 μM CP. Colorless protein was dialyzed against 0.1 M EDTA (pH 8.0) for 24 h, and then three 12-h periods against PBS (at 4 °C). Based on atomic absorption results, apo-CP in this preparation contained less than 0.05 copper atoms per molecule. The absence of trace amounts of chelating agents was proven by special control experiments [7]. The percent of CP fragments was calculated by a densitometric analysis performed using ImageJ software (Table 1). Purity of CP and composition of CPprot fragments is presented on Figure S1 in Supplementary file. 

During this study, the authors used synthetic amino acid sequences purified by high-performance liquid chromatography (NPF Verta, Saint-Petersburg, Russia). The amino acid sequences of the peptides used in this study are presented in Table 2.

Lactoferrin (LF) was isolated from the breast milk of healthy volunteers using ion-exchange chromatography with CM-Sepharose, and by gel-filtration with Sephadex G-100 Superfine [10]. Recombinant human tumor necrosis factor-α (TNF-α) was from PeproTech (Rocky Hill, NJ, USA). 

### 2.2. Ethics Statement

The authors prepared neutrophils from the blood of healthy volunteers. Blood was collected via venous puncture, as approved by the Ministry of Public Health Service of the Russian Federation. Experimental and subject consent procedures were approved by the Institutional Ethics Committee of the A. N. Belozersky Institute of Physico-Chemical Biology, Moscow State University. 

### 2.3. Human Neutrophil Isolation

Regarding neutrophil preparations, PMNLs were isolated from freshly drawn citrate-anti-coagulated donor blood by standard techniques, as previously described [17]. The authors prepared leukocyte-rich plasma through the sedimentation of red blood cells (RBCs) using 3% dextran T-500 at room temperature. Granulocytes were then purified by centrifugation of leukocyte-rich plasma using Ficoll-Paque (density 1.077 g/mL), followed by hypotonic lysis of the remaining RBCs. PMNLs were washed twice with phosphate buffered saline (PBS), resuspended at 10^7^/mL (purity 96–97%, viability 98–99%) in Dulbecco’s PBS containing 1 mg/mL glucose (without CaCl_2_), and stored at room temperature. 

### 2.4. Reactive Oxygen Species (ROS) Analysis 

#### 2.4.1. Assessment of Extracellular Superoxide Radicals by Cytochrome C Reduction

Extracellular production of superoxide anion (·O_2_^−^ was measured by the SOD-inhibitable reduction of ferricytochrome c (Cyto C) [18,19]. Briefly, human neutrophils (10^6^/mL) were cultured in HBSS/HEPES containing 25 µM Cyto C and test substances in fibrinogen-coated 24-well plates for 30 min at 37 °C in 5% CO_2_. To create positive controls, oxidative activity was stimulated with phorbol 12-myristate 13-acetate (PMA) at a concentration of 1 nM. The assay was performed in the presence and absence of Cu/Zn-SOD (30 U/mL). The rate of Cyto C reduction was measured by recording the differences between absorption values at 550 and 531 nm. To control cell-free experiments, the test compounds were incubated with 25 µM Cyto C under the same conditions. The hypoxanthine (HX, 100 µM)—xanthine oxidase (XO, 0.005 U/mL) system was used to generate superoxide anions [20,21]. The rate of Cyto C reduction was measured as indicated previously herein. 

#### 2.4.2. Assessment of Intracellular ·O_2_^−^ Formation Using Dihydroethidium

Intracellular ·O_2_^−^ production was monitored by red fluorescence measurements on products of the reaction between superoxide and dihydroethidium (DHE). The cells (10^6^/mL) were incubated in HBSS/HEPES supplemented with 10 µg/mL DHE and test substances. To create positive controls, oxidative activity was stimulated using phorbol 12-myristate 13-acetate (PMA) at a concentration of 1 nM. PMNLs were cultured for 60 min at 37 °C in 5% CO_2_. Then, samples were analyzed using a Cytomics FC 500 Flow Cytometry System. Fluorescence was collected by photomultipliers at 590 nm and 620 nm [6]. Representing each acquisition, at least 10,000 events were collected within 90 s of measurement. 

#### 2.4.3. Assessment of Intracellular ROS Formation Using 2′,7′-Dichlorofluorescein-Diacetate (DCFH-DA)

Intracellular ROS formation was monitored by fluorometric measurements of green fluorescence (excitation: 485 nm, emission: 535 nm) after incorporation of carboxy-H_2_DCF-DA (5 µM; ThermoFisher Scientific, Waltham, MA, USA), in accordance with the manufacturer’s protocol. Briefly, human neutrophils were incubated with 5 μM carboxy-H_2_DCF-DA for 60 min at room temperature which was followed by washing with PBS. Cells were seeded in fibrinogen-coated 24-well plates (10^6^/mL, in HBSS/HEPES) and treated with the investigated compounds for 60 min at 37 °C in 5% CO_2_. To control cell-free assays, 10 μM carboxy-H_2_DCF-DA was incubated with intact CP, CPprot, and apo-CP (0.05–2 μM) in PBS for 30 min at 37 °C. The increase in fluorescence was then monitored with a fluorometric plate reader CLARIOstar (BMG Labtech, Ortenberg, Germany), exciting the sample at 480 nm and reading at 530 nm.

### 2.5. Apoptosis Detection by Flow Cytometry

#### 2.5.1. Double Alexa Fluor-Conjugated Annexin-V and Propidium Iodide Labeling

To detect phosphatidylserine externalization as one of the earliest indicators of apoptosis, and to ensure that the mechanism of observed cell death was through apoptosis and not necrosis, neutrophils were double-stained with Alexa Fluor-conjugated Annexin V and propidium iodide (PI). PMNLs were suspended at a density of 1 × 10^6^ cells/mL in RPMI-1640 medium supplemented with sodium bicarbonate, 2 mM L-glutamine, and 20 mM HEPES, with or without the tested compounds. Cells were cultured in 24-well plates for 5 and 16–20 h at 37 °C in a 5% CO_2_ incubator. Following incubation, cells were collected by centrifugation at 270× *g* and washed once with cold PBS. Cell pellets were resuspended in 100 μL HBSS/HEPES containing 2.5 μL Annexin V-Alexa Fluor^®^ 488 commercial solution as specified in the manufacturer's instructions. Following 10 min on ice, 300 μL PI solution (10 µg/mL HBSS/HEPES) was added for 5 min. The samples were analyzed using a Cytomics FC 500 Flow Cytometry System (Beckman Coulter, Krefeld, Germany) with CXP software. Fluorescence was collected by photomultipliers at 525 nm (Annexin V-Alexa Fluor^®^ 488) and 620 nm (PI). Representing each acquisition, at least 10,000 events were collected within 90 s of measurement. 

#### 2.5.2. Assessment of DNA Fragmentation by PI Staining of Hypo-Diploid Nuclei

PMNL apoptosis was assessed according to the percent of cells with hypo-diploid DNA content (SubG1 subpopulation), using the technique described by Nicoletti et al. [22]. Briefly, PMNLs were incubated as described previously herein. Upon reaching the end of the desired period, cells were harvested, supplemented with ice-cold 0.05% BSA in PBS and collected by centrifugation at 270× *g*, which was followed by permeabilization in cold hypotonic PI solution (20 µg/mL PI and 0.2 mg/mL RNase in 0.1% triton X-100 in 0.1% sodium citrate). The tubes were placed at 4 °C in the dark for 10–15 min before flow-cytometric analysis using a Cytomics FC 500 System and a 620-nm long-pass filter.

#### 2.5.3. Terminal Deoxynucleotidyl Transferase dUTP Nick End Labeling (TUNEL)

Neutrophils apoptosis was detected and quantified by measuring fragmented DNA by catalytically incorporating fluorescein-12-dUTP at 3′-OH DNA ends using the recombinant terminal deoxynucleotidyl transferase (rTdT), according to the manufacturer's instructions (DeadEnd™ Fluorometric TUNEL System, Promega, Madison, WI, USA). PMNLs were incubated as described. Following incubation, cells were collected by centrifugation at 270× *g*, washed once in cold PBS, resuspended in PBS and fixed with methanol-free paraformaldehyde (final concentration 1%) for 10 min at 37 °C. Next, PMNLs were centrifuged at 270× *g*, washed once in PBS, and permeabilized with 70% ice-cold ethanol for 30 min on ice. Fixed and permeabilized PMNLs were collected, washed once in PBS and resuspended in Equilibration buffer for 5 min. Probes were then centrifugated and pellets were resuspended in rTdT Incubation buffer comprising both nucleotide mix and rTdT enzyme. Probes were incubated for 60 min at 37 °C in the dark. The reaction was terminated by adding SSC buffer followed by washing with PBS. Representing negative controls, fixed and permeabilized cells were treated with Incubation buffer including only the nucleotide mix and not containing rTdT. Labeled PMNLs were analyzed using CytoFLEX Flow Cytometer system (Beckman Coulter, Krefeld, Germany) measuring green fluorescence of fluorescein-12-dUTP at 525 nm.

### 2.6. Statistics

Results are reported as mean ± SEM. Analysis of the statistical significance was evaluated using a one-way ANOVA with an appropriate post-test, using GraphPadPrism6 software. *p* values of less than 0.05 were considered significant. Data were compared against the unstimulated control and the TNF-α-stimulated samples.

## 3. Results

### 3.1. Intact and Partially-Proteolyzed Ceruloplasmin Have a Dual Effect on the Oxidant Status of Resting and TNF-α-Stimulated Neutrophils

**Figure 1:**
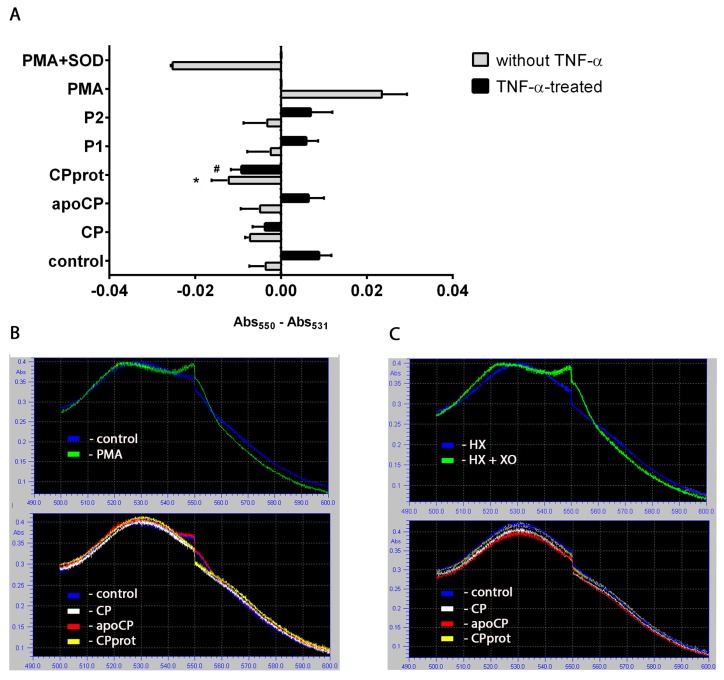
Effects of ceruloplasmin (CP) and its derivatives on superoxide generation by neutrophils and cell-free cytochrome c (Cyto C) reduction. (**A**) Polymorphonuclear leukocytes (PMNLs; 10^6^/mL) were supplemented with Cyto C and cultured with 0.5 µM CP, 0.5 µM apo-CP, 0.5 µM CPprot (proteolyzed ceruloplasmin), or 20 µM peptides (P1 or P2), with or without 25 ng/mL TNF-α. Phorbol 12-myristate 13-acetate (PMA; 1 nM) and 30 U/mL superoxide dismutase (SOD) were used as positive and negative controls, respectively. Cells were incubated in 24-well plates for 30 min at 37 °C. The rate of Cyto C reduction was measured by the change in differences between absorptions at 550 and 531 nm. Values represent the means ± SEM of Δ (550/531) of duplicate cultures from six independent experiments; *, # *p* < 0.05 compared to control (medium) and TNF-α-treated PMNLs, respectively (one-way ANOVA followed by Holm–Šídák multiple comparison tests). (**B**) Representative spectra recorded at 600–500 nm of Cyto C reduction in PMNL incubations with apo-CP, CP, CPprot, and PMA. **C.** Cyto C was incubated with 0.5 µM CP, 0.5 µM apo-CP, 0.5 µM CPprot or with 100 μM HX + 0.005 U/mL XO following spectra recording as above.

To identify the test compounds that affect PMNL redox status, the authors employed several approaches, considering the specificity and limitations of each probe used to analyze oxidative burst in neutrophils [23,24,25]. When measured by SOD-dependent cytochrome *c* reduction, unlike apo-CP or synthetic peptides, CP and especially CPprot reduced spontaneous, and completely extinguished TNF-α-induced extracellular ·O_2_^−^ generation (Figure 1A,B). Featured in control cell-free experiments tested compounds did not affect reduced Cyto C accumulation (Figure 1C).

**Figure 2:**
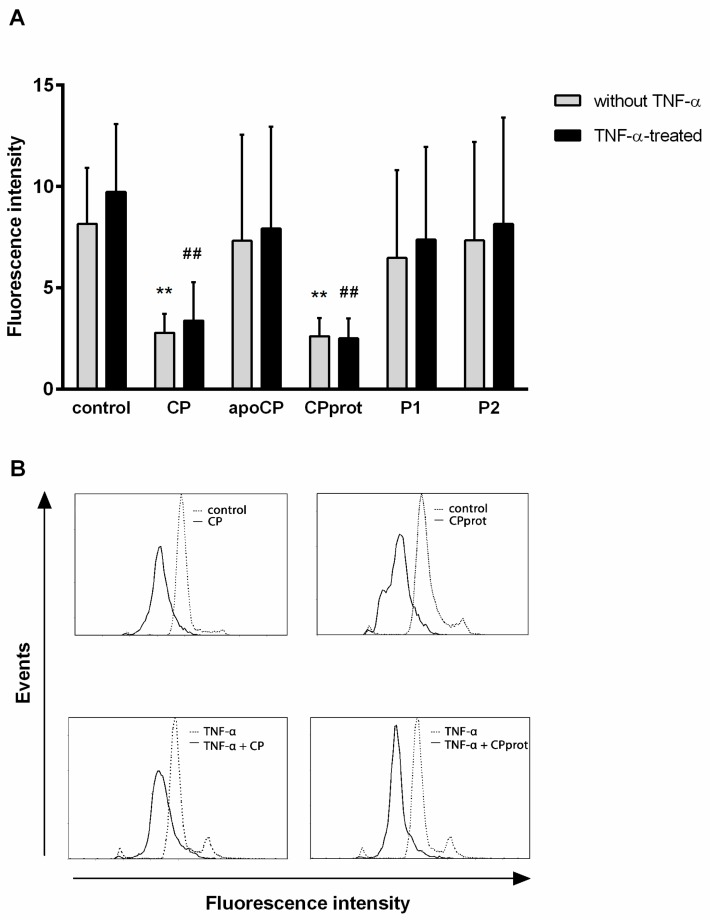
Effects of ceruloplasmin (CP) and its derivatives on intracellular superoxide generation by neutrophils. Dihydroethidium (DHE)-stained polymorphonuclear leukocytes (PMNLs) were cultured with 0.5 µM CP, 0.5 µM apo-CP, 0.5 µM proteolyzed CP (CPprot), or 20 µM peptides (P1 or P2), with or without 25 ng/mL TNF-α for 60 min at 37 °C, which was followed by flow cytometric measurements of red fluorescence. (**A**) Values represent the means ± SEM of fluorescence intensities, arbitrary units of duplicate cultures from four independent experiments; **, ^##^ *p* < 0.01, compared to the control (medium) and TNF-α-treated PMNLs, respectively (one-way ANOVA followed by Holm–Šídák multiple comparison tests). (**B**) Representative histograms of PMNL distribution according to accumulation of DHE oxidation products.

CP or CPprot were found to reduce intracellular ·O_2_^−^ levels, similar to that observed in unstimulated cells, with the addition of TNF-α, whereas the other enzyme derivatives did not exhibit any reliable effect (Figure 2). This was shown by the measurement of DHE oxidation since this probe is known to be sensitive and responsive to ·O_2_^−^ [26]. These facts confirm and supplement previous data [7].

**Figure 3:**
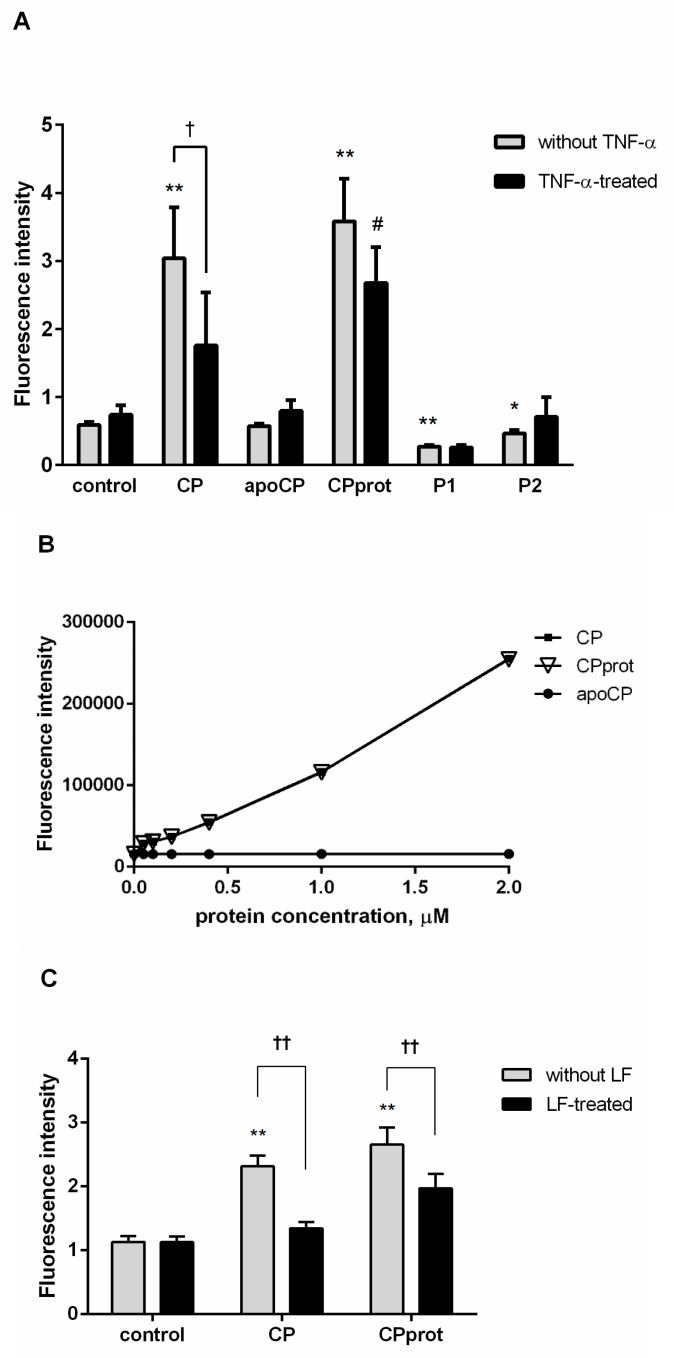
Effects of ceruloplasmin (CP) and its derivatives on intracellular reactive oxygen species (ROS) generation by neutrophils. H_2_DCF-DA stained polymorphonuclear leukocytes (PMNLs) were cultured with 0.5 µM CP, 0.5 µM apo-CP, 0.5 µM CPprot (proteolyzed CP), or 20 µM peptides (P1 or P2), with or without 25 ng/mL TNF-α (**A**) or 1 µM lactoferrin (LF) **(C)** for 60 min at 37 °C followed by fluorometric measurements of green fluorescence. (**B**) H_2_DCF-DA-oxidase activity of intact CP, CPprot (0.05–2 µM) in cell-free assay. Apo-CP was used as a control. Values represent the means ± SEM of fluorescence intensities, arbitrary units of duplicate cultures from six independent experiments; * *p* < 0.05, ** *p* < 0.01, compared to the control (medium); ^#^ *p* < 0.05, compared to TNF-α-treated PMNLs; ^†^ *p* < 0.05, ^††^ *p* < 0.01 for pairs of data compared (one-way ANOVA followed by Holm–Šídák multiple comparison tests).

Examining the oxidative status of neutrophils with cell-permeable H_2_DCF-DA showed a significant increase in the fluorescence emission of the DCF oxidation product when CP or CPprot were added to PMNLs. Concurrently, the synthetic peptides P1 and P2 weakly inhibited the generation of intracellular oxidants (Figure 3A). The addition of TNF-α by itself had a negligible effect on neutrophil redox status. However, it contributed to a decrease in the influence of CP and CPprot on oxidant formation to some degree (Figure 3A). 

Regarding control cell-free experiments, H_2_DCF-DA-oxidase activity of CP was confirmed. Intact CP and CPprot oxidized 2′,7′-dichlorofluorescin diacetate in a dose-dependent manner; however, apo-CP did not demonstrate such activity (Figure 3B). Considering this fact, additional experiments were carried out in which PMNLs were treated with copper sulfate (CuSO_4_) at concentrations of 1–3 µM. The addition of inorganic copper did not lead to the accumulation of intracellular H_2_DCF oxidants (data not shown). This led the authors to consider the revealed action as a biological effect of intact and proteolyzed ceruloplasmin, rather than just a product of free copper ions released from these compounds.

Since reactive nitrogen species are known to be possible H_2_DCF oxidants [27], additional experiments using DAF-2 DA, a cell permeable probe for the measurement nitric oxide synthase activity, were performed. These assays did not reveal any effect of the investigated compounds on nitric oxide levels.

When TNF-α is added, endogenous LF release occurs [28], which helps to stabilize the enzyme, and thereafter retain copper. Indeed, the addition of exogenous LF inhibited oxidant formation induced by CP and CPprot (Figure 3C).

Considering the data indicating that neutrophil life span is dictated by the redox status of the cell [29], we performed experiments to explore whether CP derivatives themselves or in conjunction with TNF-α affect PMNL apoptosis.

### 3.2. CP and Its Derivatives Inhibit Spontaneous PMNL Apoptosis

**Figure 4:**
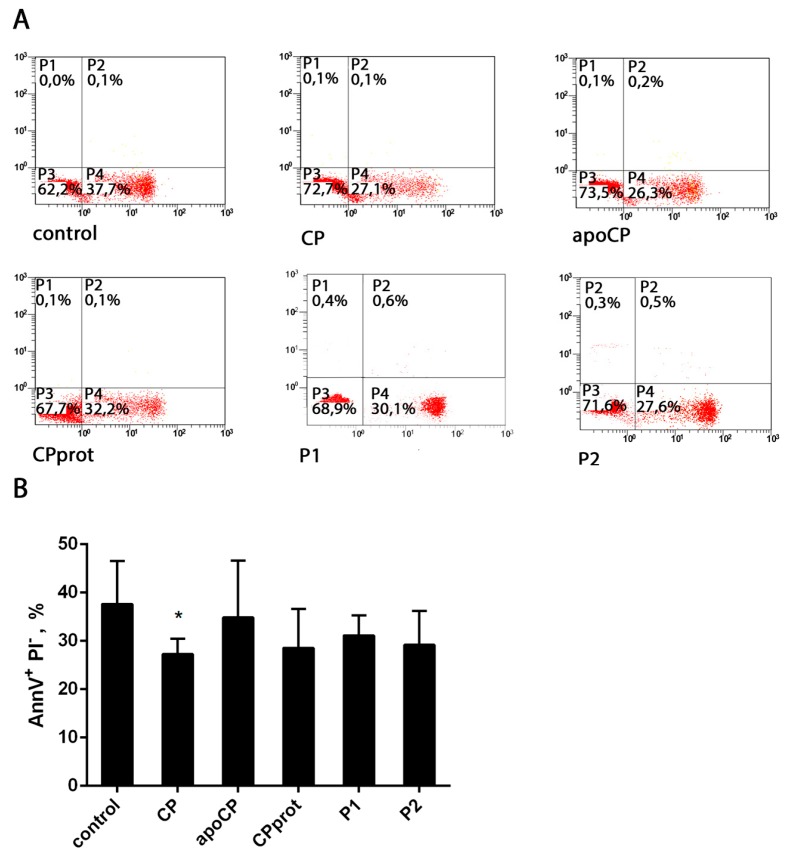
Spontaneous apoptosis of human neutrophils after exposure to ceruloplasmin (CP) or its derivatives. Polymorphonuclear leukocytes (PMNLs; 10^6^/mL) were cultured for 20 h at 37 °C without additives or with 0.5 µM intact CP, 0.5 µM apo-CP, 0.5 µM CPprot (proteolyzed CP), or 20 µM peptides (P1 or P2). The percentage of Annexin V- or propidium iodide (PI)-positive cells was determined by flow cytometry. (**A**) Representative dot plots for phosphatidylserine externalization by apoptotic cells, as well as the proportions of viable (region P3), early apoptotic (region P4), and late apoptotic and necrotic cells (regions P2 and P1) are indicated. (**B**) Presented are pooled data with the percentage of apoptotic cells. Values indicate the mean ± SEM of duplicate cultures from three independent experiments; * *p* < 0.05, compared to control (one-way ANOVA followed by Holm–Šídák multiple comparison tests).

PMNLs were incubated with no additives or in the presence of intact CP, apo-CP, CPprot, or peptide P1 and P2, corresponding to fragments of CP. Subsequently phosphatidylserine exposure and plasma membrane integrity loss were analyzed by double Alexa Fluor-conjugated Annexin-V and PI labeling. Regarding control and test samples, a negligible number of necrotic PMNLs was detected, even after prolonged (20 h) culturing (Figure 4A).

**Figure 5:**
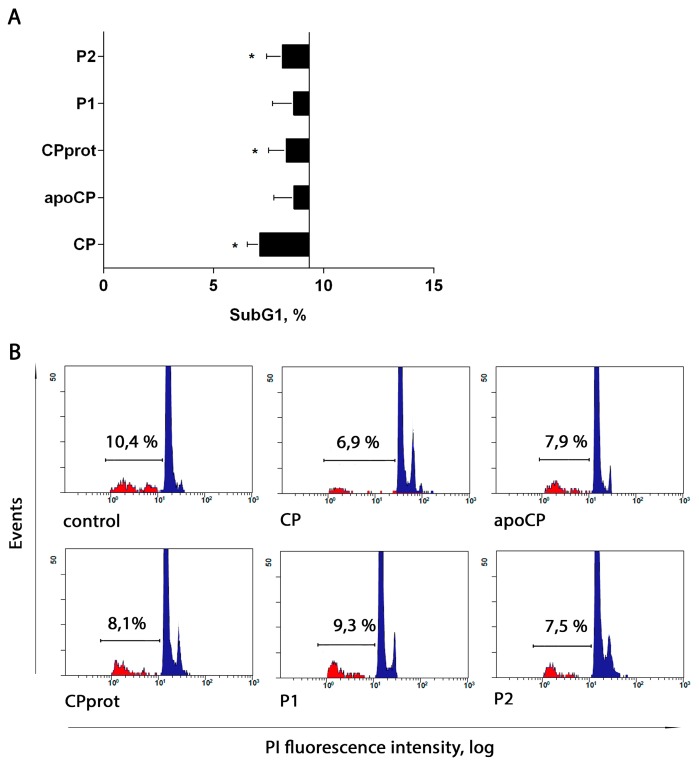
Effect of ceruloplasmin (CP) and its derivatives on spontaneous neutrophil apoptosis. Polymorphonuclear leukocytes (PMNLs; 10^6^/mL) were cultured at 37 °C with 0.5 µM intact CP, 0.5 µM apo-CP, 0.5 µM CPprot (proteolyzed CP), or 20 µM peptides (P1 or P2) for 20 h and assayed for the proportion Sub G1 PMNLs by flow cytometry. (**A**) Apoptosis levels induced by the indicated compounds. Bars begin at the control line (which corresponds to 9.3 ± 0.77%), values indicate the mean ± SEM of duplicate cultures from six independent experiments; * *p* < 0.05, compared to control (one-way ANOVA followed by Holm–Šídák multiple comparison tests). (**B**) Representative histograms of nuclear DNA fragmentation.

**Figure 6:**
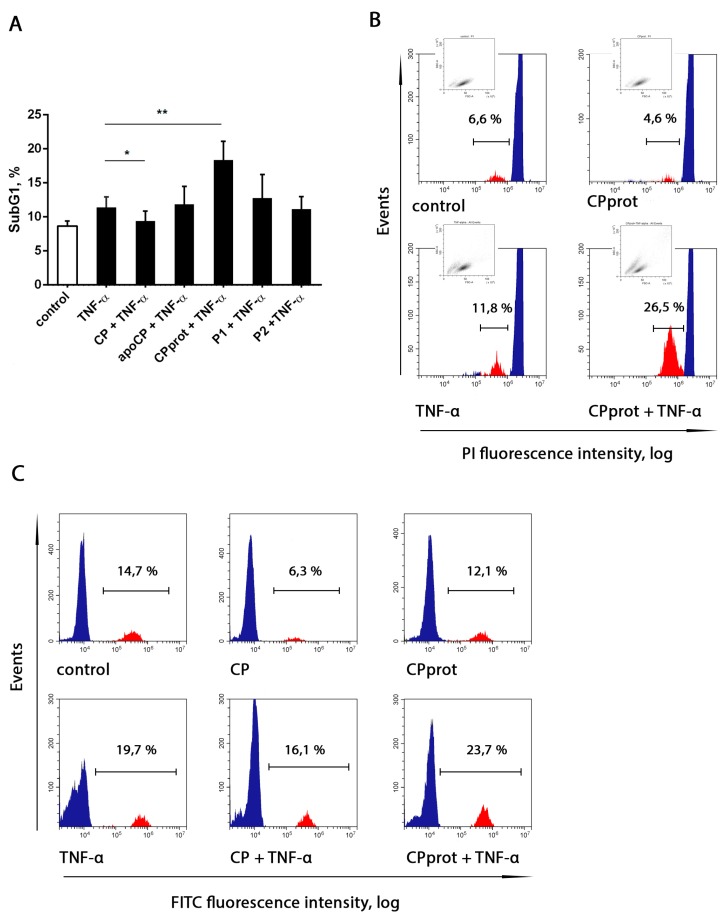
Neutrophil apoptosis with simultaneous exposure to ceruloplasmin (CP) and its derivatives and TNF-α. Polymorphonuclear leukocytes (PMNLs; 10^6^/mL) were cultured at 37 °C with 0.5 µM intact CP, 0.5 µM CPprot (proteolyzed CP), 0.5 µM apo-CP, or 20 µM peptides (P1 or P2), with or without 25 ng/mL TNF-α for 20 h; nuclear DNA fragmentation was assayed both by PI staining of hypo-diploid nuclei and by the detection of DNA strand breaks by TUNEL. (**A**) Presented are pooled data with the percentage of apoptotic cells. Values are the mean ± SEM of duplicate cultures from six independent experiments; * *p* < 0.05 and ** *p* < 0.01 (one-way ANOVA followed by Holm–Šídák multiple comparison tests). Representative histograms of apoptotic DNA fragmentation revealed as the SubG1-subpopulation (**B**) and as labeled with FITC-12-dUTP DNA strand breaks (**C**).

The authors found no statistically significant modulatory effects on PMNL apoptosis for CP and its derivatives with short-term incubation (5 h). However, prolonged exposure to the proteins and peptides slightly but stably suppressed spontaneous apoptosis, as revealed using both phosphatidylserine externalization detection (Figure 4) and assessment of nuclear DNA fragmentation by SubG1 PMNLs estimation (Figure 5) or TUNEL assay (Figure 6C) followed by flow cytometric analysis. When compared to that in control samples, the growth of living cells number was more pronounced for intact CP (Figure 4B and Figure 5A). 

### 3.3. Proteolyzed Ceruloplasmin Enhances the Pro-Apoptotic Effect of TNF-α, Whereas the Intact Protein Retains Pro-Survival Properties

It is known that TNF-α is one of the cytokines that are part of the acute phase reaction, and this molecule plays a critical role in the pathogenesis of chronic inflammatory diseases by eliciting a broad spectrum of cellular responses, including proliferation, differentiation, and apoptosis. Considering that, the authors investigated whether CP, or its derivatives, and TNF-α, added at a potentially apoptogenic dose (25 ng/mL) simultaneously with the tested compounds, mutually influence the apoptosis-modifying properties of each other. Notably, in these experiments, TNF-α affected neutrophil viability in an irregular manner. Treatment with this cytokine sharply enhanced PMNL apoptosis at early times of incubation. However, with prolonged incubation, the pro-apoptotic activity of TNF-α was markedly diminished, as determined by measuring Annexin-V-positive neutrophils (data not shown) and Sub G1 fractions (Figure 6). These results are generally consistent with data from other research groups confirming the existence of different pathways in TNF-α signaling [30].

Upon short-term incubation, simultaneous addition of the tested compounds and TNF-α did not alter the percentage of Annexin-V-positive neutrophils, compared to that in samples treated with TNF-α alone. When neutrophil apoptosis was assessed after prolonged incubation, the authors revealed a significantly augmented TNF-α apoptogenic effect with the simultaneous addition of the cytokine and CPprot, and a slight pro-survival effect with the CP/TNF-α combination (Figure 6). Neither apoCP nor synthetic peptides had any effect on TNF-α-induced PMNL apoptosis.

## 4. Discussion

This study demonstrates that, in contrast to other CP derivatives investigated, the intact enzyme and the product of its partial proteolysis have a pronounced effect on PMNL oxidative status. CP and CPprot reduced extra- and intracellular ·O_2_^−^ levels arising both spontaneously and with TNF-α stimulation. Concurrently, the authors observed a sharp increase in the fluorescence of the oxidation product of H_2_DCF, used for nonselective quantitation of intracellular oxidants. Regarding control, cell-free experiments, the authors confirmed the H_2_DCF-DA oxidase activity of CP and CPprot (Figure 3B). It was suggested that both effects are associated with the presence of copper in both compounds. Indeed, apo-CP did not prevent the formation of superoxide or the oxidation of DCFH-DA. The mechanisms of superoxide elimination by CP are not completely clear, but the most plausible is the hypothesis that this ability is inextricably linked to the oxidative activity of copper atoms [31,32]. When reduced by superoxide, Cu+ catalyzes hydroxyl radical formation; both oxidized and reduced copper ions catalyze and amplify the formation of ROS, thus affecting intracellular and tissue structures. Estimating the oxidant status using DHE and 2′,7′-dichlorofluorescin diacetate, the authors obtained opposite results: the addition of intact or proteolyzed ceruloplasmin led to a diminution in oxidized DHE fluorescence (Figure 2), but dramatically increased the DCF emission (Figure 3). Such data inconsistency indirectly confirms precisely this mechanism. Moreover, the resulting formation of oxidants leads to structural destabilization of CP, and possibly CPprot, resulting in increased lability of copper associated with both compounds [33,34]. The ability of CP to exhibit both anti-oxidant and pro-oxidant properties persists after its partial proteolysis. Moreover, proteolytic degradation promotes some increase in pro-oxidant activity. Obviously, under conditions of inflammation, when the concentration of CP and, accordingly the products of its hydrolysis, increase, mechanisms regulating pro-oxidant activity must exist. During these experiments, the addition of TNF-α had an inhibitory effect on the formation of intracellular oxidants. This might be due to the stabilization of CP and CPprot by LF, which is released from granules under the control of TNF-α. 

Intracellular oxidant accumulation often triggers apoptotic pathways; in addition, copper ions which become more accessible due to the oxidative damage of intact and proteolyzed CP might have detrimental intracellular effects. Based on this, and due to similarities between CP and CPprot in terms of their influence on the oxidative status of neutrophils, it was expected that these compounds would identically affect PMNL apoptosis. However, the unidirectional ability to inhibit both spontaneous and TNF-α induced apoptosis was observed only for intact CP, whereas the regulation of apoptosis by derivatives of CP switched from inhibition to activation with the addition of TNF-α. The authors emphasize once again that the revealed effects are the bioactivity of CP and CPprot, since inorganic copper, when added to the cells in relevant concentrations, did not exert the same influence on PMNL oxidative or apoptotic status (data not shown).

Although the molecular mechanisms of these effects are beyond the scope of this study, the authors assume that proteins of neutrophil origin are at the foreground. 

MPO is an oxidative enzyme that is capable of inducing oxidative, halogenative, and nitrosative stress in vivo [35]. Hypochlorous acid (HOCl) is the major oxidant formed by MPO under physiological conditions [36]. Supplemental to its microbicidal action, HOCl influences the functions of PMNL by modifying proteins, lipids, and DNA, thereby reducing the lifespan of neutrophils themselves [37] as well as neighboring cells [38]. Additionally, MPO was previously shown to be a powerful auto- and paracrine mediator, which can both delay [39] and initiate apoptosis [40,41]. 

TNF-α plays a critical role in modulating acute and chronic inflammation, largely due to its ability to fine-tune neutrophil functions [28,42,43] and lifespan [44,45]. Specifically, this cytokine markedly stimulates MPO synthesis and secretion. MPO-dependent HOCl is known to accumulate during long incubations of neutrophils with TNF-α [46] and is facilitated by cytokine-induced cellular adhesion [47]. TNF-α induced degranulation leads to inter alia LF release from secondary granules [28,48]. 

CP is known as a physiological inhibitor of MPO pro-oxidative activity [49]. The ability of CP to inhibit the formation of MPO-derived oxidants is obviously another possible contributor to the anti-apoptotic activity of intact CP observed in these assays. It should be noted that the processes in question occur during long incubations. The oxidative status of PMNLs was evaluated in short-term experiments. Additionally, it is known that hydrophilic H_2_DCF, produced from H_2_DCF-DA by intracellular cleavage, is unable to penetrate membranes including lysosomal [50]; hence, the method used characterizes the oxidase status of the cytosol, but not the granules. Increased oxidation of H_2_DCF in cell and cell-free assays by CP indirectly shows the possibility of CP endocytosis by neutrophils. Therefore, the contradiction regarding the previous assumption and the effect of CP relating to oxidative status (based on the data) seems to be misleading.

CP and its derivatives differ in their ability to inhibit MPO peroxidase activity [51]. The limited proteolysis of CP was shown to abrogate its capacity to inhibit the peroxidase activity of MPO [14]. The inability of all compounds except holo-CP to prevent TNF-α-induced apoptosis was probably due to decreased affinity of CP derivatives for the peroxidase enzyme. It also has been established that CP influences the activities of serprocidins of neutrophil-origin, including proteinase 3 (PR3) [52]. The latter suggests one possible mechanism of the spontaneous death program in aging neutrophils [53]. One can assume that the suppression of spontaneous apoptosis, observed with the compounds studied, occurs through the inhibition of PR3, and that the inhibitory effect would depend on the integrity of the copper protein.

The release of LF from TNF-α-treated cells [28] can also contribute to the observed influence of CP and its derivatives on neutrophil apoptosis. First, LF is the most active protector of CP [54]. Oxidative stress and ROS disrupt copper binding to CP, thereby impairing its normal function [33]. CP is easily degraded by proteases [55,56]. However, the integrity of the CP molecule is important for the function of this enzyme. Antioxidant properties such as GSH-dependent peroxidase activity [57] and the inhibition of the chlorinating and peroxidase activity of MPO [14] are lost after proteolytic degradation. Additionally, the enzyme disintegration process itself is accompanied by hydroxyl radical generation [54]. It could be argued that upon TNF-α stimulation, interactions between LF and CP protect the anti-oxidant properties of the latter and prevents excessive OH·-formation. 

Additionally, LF inhibits neutrophil apoptosis such that its pro-survival effect is dependent on its iron saturation status; specifically, iron-saturated LF is incapable of affecting neutrophil life span [58]. CP and LF mutually affect the function of each other. LF promotes the Fe^2+^ binding and ferroxidase activity of CP [59]. Subsequently, CP stimulates iron incorporation into apo-LF [59]. LF can incorporate ferric ions produced during ferroxidase reactions catalyzed by CP. Therefore, the oxidation of pro-oxidant ferrous ions catalyzed by CP should increase the iron saturation status of LF and reduce its ability to affect neutrophil apoptosis. This becomes particularly important for proteolyzed CP. Mentioned previously, the latter fails to inhibit the peroxidase activity of MPO [14] but retains its ferroxidase activity, thus, suppressing the anti-apoptotic properties of LF. These results showed that CPprot facilitates a TNF-α-induced apoptotic death program in neutrophils, and it is assumed that the aforementioned mechanism is a prerequisite for this event. 

It was found that partial proteolysis dramatically reduces the inhibitory function of MPO on CP without disturbing its ferroxidase activity [14]. CPprot cannot inhibit the peroxidase activity of MPO [14], but retains ferroxidase activity. LF can incorporate ferric ions produced during the ferroxidase reaction catalyzed by CP; for example, LF reduces the oxidation potential of pro-oxidant iron ions [10]. Therefore, oxidation of pro-oxidant ferrous ions catalyzed by CP should increase the iron saturation status of LF and reduce its ability to affect neutrophil apoptosis. 

The generation of reactive oxygen, halogen, and nitrogen species by neutrophils has been implicated in the onset and progression of several disorders [35,60]. Although defense against oxidative stress has been demonstrated for CP, many studies have indicated that this protein is able to act as a powerful pro-oxidant factor. Being effective superoxide radical scavengers, these results indicate that, not only intact, but also partially proteolyzed CP, indeed induces an immediate sharp increase in other intracellular oxidants, at least in experimental conditions. Regarding conditions of oxidative stress, CP-derived peptides also possess pro-oxidant activities and potentiate phorbol myristate acetate-induced ROS formation [61]. Contrary to the unidirectional effect on PMNL oxidant status, intact and partially proteolyzed CP acted differently on delayed apoptosis. The inhibition of apoptosis observed in the presence of acute phase CP occurred whether or not PMNLs were exposed to TNF-α. The mode of apoptosis regulation by CPprot, as well as other products of enzyme hydrolysis, switches from inhibition to activation in the presence of TNF-α. Proteolytic degradation of CP is one characteristic of inflammatory processes. Analysis of the synovial fluid of patients with rheumatoid arthritis revealed that CP is proteolytically degraded to a variable extent during this inflammatory process [14]. The authors propose that this event represents a potential mechanism associated with the activation of neutrophil apoptosis. The net effect of CP on neutrophil survival no doubt reflects a balance between the activities of numerous inflammation factors, not only TNF-α. The nature and mechanisms of the effects of CP on the non-enzymatic activity of MPO are poorly understood and should be the subject of further research. Nevertheless, it is clear that a holo-CP and the product of its physiological decay are directly involved in the fine regulation of inflammatory processes. To the authors’ knowledge, this is the first study demonstrating the regulation of neutrophil apoptosis by CP and its derivatives.

## Figures and Tables

**Table 1:** Quantitative evaluation of protein preparations.

Compound	A_610_/A_280_	M (Percentage), kDa
Intact CP	>0.050	132 (94%), 116 (2%), 19 (2%)
CPprot	>0.049	116 (18%), 72 (16%), 67 (16%), 52 (16%), 19 (34%)
Apo-CP	<0.0001	132 (94%), 116 (2%), 19 (2%)

**Table 2:** Amino acid sequences of the peptides used in this study.

Abbreviation	Amino Acid Sequence	Molecular Mass (Da)
P1	RPYLKVFNPR (883–892)	1290
P2	RRPYLKVFNPRR (882–893)	1600

## Supplementary Materials

Supplementary materials are available on line www.mdpi.com/2073-4409/7/1/8/s1.
