# Supplementary material for: Fine Regulation of Neutrophil Oxidative Status and Apoptosis by Ceruloplasmin and Its Derivatives
Source: Cells. 2018 Jan 12;7(1):8. doi: 10.3390/cells7010008 (PMC5789281; doi:10.3390/cells7010008)

Fig. S1. SDS-PAGE analysis of intact human CP (1), human CP after 2 hrs of limited proteolysis by thrombin (100:1, w/w) and benzamidine-agarose chromatography - CPprot (2) , and apo-CP (3). Coomassie R-250 staining, 20 μg loaded.

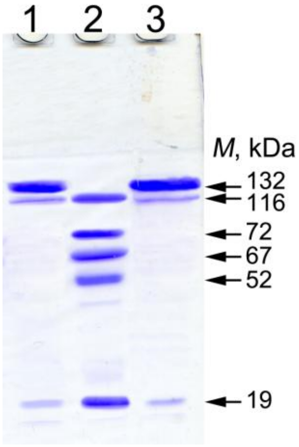

Supplement: Supplementary file 1 [file cells-07-00008-s001.pdf]
